# Effectiveness and perinatal outcomes of history-indicated, ultrasound-indicated and physical examination-indicated cerclage: a retrospective study

**DOI:** 10.1186/s12884-022-04557-7

**Published:** 2022-03-17

**Authors:** Ceren Golbasi, Hakan Golbasi, Burak Bayraktar, Baris Sever, Tayfun Vural, Atalay Ekin

**Affiliations:** 1 Department of Obstetrics and Gynecology, Faculty of Medicine, Izmir Tinaztepe University, Izmir, Turkey; 2 Department of Obstetrics and Gynecology Division of Perinatology, University of Health Sciences Tepecik Training and Research Hospital, Izmir, Turkey; 3 Department of Obstetrics and Gynecology, University of Health Sciences Tepecik Training and Research Hospital, Izmir, Turkey

**Keywords:** Cervical cerclage, Cervical insufficiency, Preterm birth, Perinatal outcome

## Abstract

**Objective:**

To evaluate the effectiveness and perinatal outcomes of cerclage procedure according to indication.

**Methods:**

The pregnancy and neonatal outcomes of the patients who underwent cerclage with the diagnosis of cervical insufficiency between January 2016 and December 2020 were retrospectively analyzed. Patients were categorized into three groups: a history-indicated group, an ultrasound-indicated group and a physical examination-indicated group.

**Results:**

Seventy-three patients who underwent cerclage were included in the study. Of these, 41 (56.2%) had history-indicated, 17 (23.3%) had ultrasound-indicated and 15 (20.5%) had physical examination-indicated cerclages. Compared to history- and ultrasound-indicated cerclage group, duration from cerclage to delivery (18.6 ± 6.9 weeks vs 17.8±5.9 weeks vs 11 ± 5.3 weeks, *p* = 0.003) was significantly lower and delivery < 28 weeks (9.8% vs 5.9% vs 33.3%, *p* = 0.042) and delivery < 34 weeks of gestation (26.8% vs 11.8% vs 60%, *p* = 0.009) were significantly higher in physical examination-indicated cerclage group. In physical examination-indicated cerclage, compared with history- and ultrasound-indicated cerclage low birth weight, low APGAR score, neonatal intensive care unit admission and neonatal mortality were higher, although not statistically significant (*p* > 0.05).

**Conclusion:**

Pregnant women who underwent physical examination-indicated cerclage had higher risks for preterm delivery < 28 weeks and < 34 weeks than history- and ultrasound–indicated cerclage.

## Introduction

Preterm birth is the leading cause of neonatal morbidity and mortality worldwide and accounts for approximately 12% of all pregnancies [1]. There are many conditions in the etiology of preterm birth and among these reasons, cervical insufficiency has a significant contribution [2]. 20% of miscarriages and extreme preterm deliveries between 16 and 27 weeks are thought to be caused by cervical insufficiency [3]. Cervical insufficiency, also known as painless early cervical dilatation that occurs in the second trimester may be due to many reasons. The structure of the cervix consists mainly of connective tissue and therefore, the cervix is insensitive to contractions. Abnormalities of this connective tissue may cause development of cervical insufficiency.

The diagnosis of cervical insufficiency is not easy due to absence of clear diagnostic criteria. The most widely used diagnostic method in non-pregnants is the passage of Hegar number 8 dilators through the cervical canal in the luteal phase without any resistance [4]. However, the most important auxiliary method for diagnosis of cervical insufficiency during pregnancy is ultrasonography [5]. According to detailed examination with ultrasonography and general characteristics of the patient, various diagnostic parameters have been defined to make a cerclage indication for the treatment of cervical insufficiency [6]. These indications can be generally divided into following 3 groups: a) History-indicated cerclage (prophylactic cerclage) b) Ultrasound-indicated cerclage c) Physical examination-indicated (emergency or rescue) cerclage.

Studies evaluating the effectiveness and complications of cerclages are limited. Investigating the effectiveness of each cerclage indication with an appropriately designed randomized control study poses clinical follow-up and ethical challenges. However, data obtained from cohort studies and case series indicate that fetal well-being and neonatal survival rates were higher in patients treated with cerclage compared to patients who managed with an observational approach [7, 8].

In this study, we aimed to evaluate the effectiveness and perinatal outcomes of cerclage in patients with cervical insufficiency for the prevention of preterm birth according to different indications.

### Materials and methods

Patients who were diagnosed with cervical insufficiency at University of Health Sciences Tepecik Training and Research Hospital, Izmir, Turkey between January 2016 and December 2020 were included in this study. Patients’ medical information was analyzed retrospectively using the data network of our center. Patients with multiple pregnancy, membrane rupture, systemic diseases, fetal congenital malformations and active preterm labor were excluded from the study. Indications for cerclage procedure were divided into three groups: 1) history-indicated cerclage 2) ultrasound- indicated cerclage and 3) physical examination-indicated cerclage. History-indicated cerclage group included those who had a history of two or more second-trimester pregnancy losses after painless cervical dilatation. Ultrasound-indicated cerclage group included patients with a history of preterm labor in their previous pregnancy and with short transvaginal cervix length (< 25 mm) in mid-trimester routine ultrasound. Physical examination-indicated cerclage group included patients with asymptomatic cervical dilatation ≥ 2 cm and cervical effacement ≥ 60% or amniotic membrane prolapse in the mid-trimester period.

Cerclage placement was planned at the end of the first trimester for history-indicated group and at the time of diagnosis for ultrasound- and physical examination-indicated group. Ultrasound- and physical examination-indicated cerclage procedures were not performed to pregnancies over 24 gestational weeks. Before placement of the cerclage, the presence of uterine contraction, abdominal pain or tenderness, fever, membrane rupture, leukocytosis (> 18.000/µL), vaginal bleeding, chorioamnionitis, placental ablatio and fetal distress were evaluated. Examinations and cerclage procedures of all patients were performed by experienced clinicians of our perinatology department. All patients underwent cerclage procedure via McDonald technique under spinal anesthesia. After disinfection of vagina and cervix with povidon iodine solution under lithotomy position, the cervix was grasped with ring forceps from both anterior and posterior lips. A 5-mm braided polyester fiber (Mersilene®) tape suture was placed circumferentially around the cervicovaginal junction. The suture was placed counterclockwise direction from 11 o’clock without entering endocervix and knot was tied at 12 o’clock position. In patients with bulging membranes and advanced cervical dilatation, a soaked sponge was introduced to cervical canal to retract the membranes beyond external cervical os. Prophylactic antibiotic (Cefazolin 1 g, IV) were administered at the time of surgery for all patients regardless of indications. Patients without symptoms such as pain, bleeding or membrane rupture following 24 h were discharged from the hospital. Hydroxyprogesterone caproate 500 mg/2 ml, IM was recommended for use once a week during pregnancy. All cerclages were removed at 37 weeks of gestation.

All study groups were compared in terms of clinical characteristics, pregnancy and neonatal outcomes. Due to improvements in survival rates of infants born at 22 gestational weeks, our center provides life-sustaining interventions for such infants after 22 weeks. Therefore, we categorized pregnancy outcomes as pregnancy loss < 22 weeks, preterm birth <28 weeks, preterm birth <34 weeks, preterm birth <37 weeks and delivery ≥37 weeks.

SPSS (Statistical Package for the Social Sciences) 22.0 program was used for the statistical analysis. Data were given as mean ± SD and n (%). Analysis of variance test was used for the comparison of variables between groups. In cases where any significant differences were detected as a result of the analysis, the homogeneity of the variances was checked to determine the groups causing such differences. If the variances were homogeneous, Scheffe test was used. If the variances were heterogeneous, Tamhane T2 test was used. Survival analysis was calculated by Kaplan–Meier and Log-Rank methods. *p* < 0.05 was considered as significant.

The study was approved by the University of Health Sciences Tepecik Training and Research Hospital Local Ethics Committee (approval number: 2019/ 15–13). The research was conducted in accordance with the 1964 Helsinki Declaration. Informed consent is not required as it is a retrospective study.

## Results

Seventy-three patients who underwent cerclage were included in the study. There were 41 (56.2%) patients in the history-indicated group, 17 (23.3%) patients in the ultrasound-indicated group and 15 (20.5%) patients in the physical examination-indicated group. The clinical characteristics of study groups were presented in Table 1. There was no significant difference between the three groups in terms of maternal age (*p* = 0.492), parity (*p* = 0.417), miscarriage (*p* = 0.350), maternal smoking (*p* = 0.647) and conization history (*p* = 0.221). Mean number of preterm birth was significantly higher in history-indicated cerclage (2.4 ± 0.6) compared to ultrasound- (1.4 ± 0.6) and physical examination-indicated cerclage (0.6 ± 0.5) and also significantly higher in ultrasound-indicated cerclage compared to physical examination-indicated cerclage. Mean number of second trimester pregnancy loss was significantly higher in history-indicated cerclage (2.3 ± 0.5) compared to ultrasound- (0.6 ± 0.5) and physical examination-indicated cerclage (0.5 ± 0.4). The mean gestational age for history- (16.1 ± 2.7 weeks) and ultrasound-indicated cerclage (18.1 ± 3.5 weeks) was significantly lower than physical examination-indicated cerclage (20.6 ± 1.5 weeks, p < 0.001). The cervical length measurement at the time of cerclage was significantly higher in history-indicated group (27.3 ± 6.9 mm) compared to ultrasound- (17.1 ± 3.4 mm) and physical examination-indicated group (17.2 ± 6.3 mm, *p* < 0.001).

**Table 1:** Clinical characteristics of the study population

	**History- indicated cerclage (*n* = 41)**	**Ultrasound- indicated** **cerclage** (***n* = 17)**	**Physical examination- indicated** **cerclage** **(*n* = 15)**	***P*** **value**
Maternal age (year)	29 ± 6	30.5 ± 5.7	28.1 ± 4.9	0.492
Adolescent pregnancy ≤ 19 year	2 (4.9%)	1 (5.9%)	1 (6.7%)	0.974
Advanced maternal age ≥ 35 year	7 (17.1%)	4 (23.5%)	2 (13.3%)	0.740
Parity				0.417
Nulliparous	13 (31.7%)	8 (47.1%)	7 (46.7%)	
Multiparous	28 (68.3%)	9 (52.9%)	8 (53.3%)	
Miscarriage	1 (0–4)	1 (0–2)	1 (0–2)	0.350
2nd trimester pregnancy loss history	2.3 ± 0.5 ^a,c^	0.6 ± 0.5 ^c^	0.5 ± 0.4 ^a^	< 0.001
Preterm birth history	2.4 ± 0.6 ^a,c^	1.4 ± 0.6 ^b,c^	0.6 ± 0.5 ^a,b^	< 0.001
Smoking	9 (21.9%)	5 (29.4%)	5 (33.3%)	0.647
Gestational age at cerclage (weeks)	16.1 ± 2.7 ^a^	18.1 ± 3.5 ^b^	20.6 ± 1.5 ^a,b^	< 0.001
Conization history	2 (4.9%)	2 (11.8%)	3 (20%)	0.221
Cervical length (mm)	27.3 ± 6.9 ^a,c^	17.1 ± 3.4 ^c^	17.2 ± 6.3 ^a^	< 0.001

Data are presented as mean ± SD or n (%). ^a^*P* < 0.05, history-indicated group versus physical examination-indicated group. ^b^*P* < 0.05, ultrasound-indicated group versus physical examination-indicated group. ^c^*P* < 0.05, history-indicated group versus ultrasound-indicated group

**Fig. 1:**
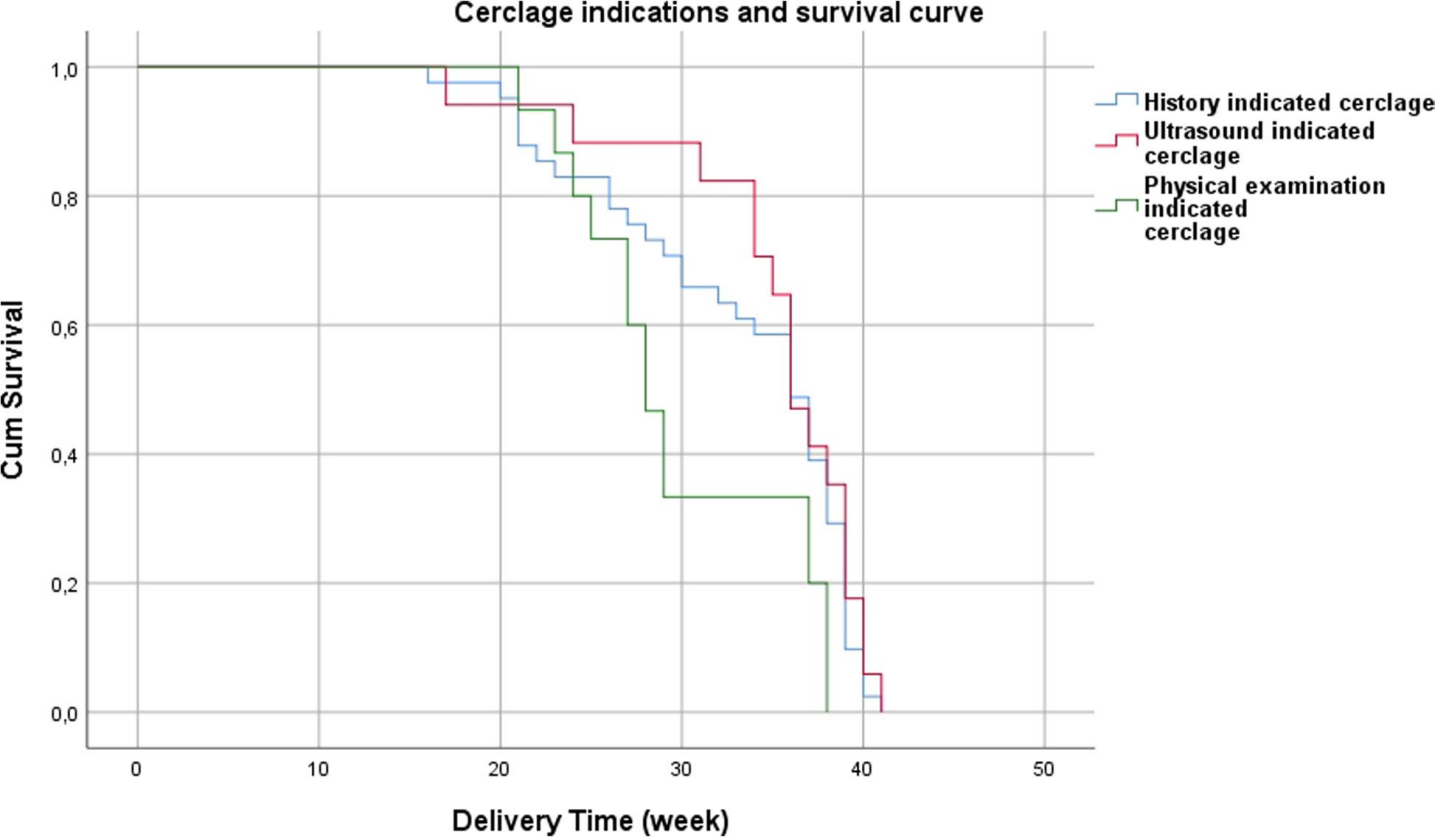
A Kaplan–Meyer survival curve of gestational age at delivery from cerclage placement until delivery, comparing patients with different indications

Pregnancy outcomes of study groups are shown in Table 2. There was no significant difference in gestational age at abortion (*p* = 0.071), gestational age at delivery (*p* = 0.102), pregnancy loss < 22 weeks (*p* = 0.861), delivery ≥37 weeks (*p* = 0.580) and type of delivery (*p* = 0.560) when compared the three groups. Preterm premature rupture of membranes (PPROM) and chorioamnionitis risk were not significantly different between groups (*p* = 0.101 and *p* = 0.673, respectively). However, the incidence of delivery < 28 weeks and delivery < 34 weeks of gestation were significantly higher (*p* = 0.042 and *p* = 0.009, respectively) and duration from cerclage to delivery was significantly lower (*p* = 0.003) in physical examination-indicated cerclage group compared to history- and ultrasound-indicated cerclage groups (Fig. 1).

**Table 2:** Pregnancy outcomes of the study population according to cerclage indications

	**History-indicated cerclage** **(*n* = 41)**	**Ultrasound-indicated** **cerclage** (***n* = 17)**	**Physical examination-indicated** **cerclage** **(*n* = 15)**	***P *value**
Gestational age of abortion	19.8 ± 2.1	17	22.3 ± 1.1	0.071
Gestational age at delivery (week)	34.9 ± 5.3	36.1 ± 4.2	31.7 ± 5.2	0.102
Duration from cerclage to delivery (week)	18.6 ± 6.9 ^a^	17.8 ± 5.9^b^	11 ± 5.3 ^a,b^	0.003
Pregnancy loss (< 22 weeks)	4 (9.8%)	1 (5.9%)	1 (6.7%)	0.861
Preterm birth < 28 weeks	4 (9.8%) ^a^	1 (5.9%) ^b^	5 (33.3%) ^a,b^	0.042
Preterm birth < 34 weeks	11 (26.8%) ^a^	2 (11.8%) ^b^	9 (60%) ^a,b^	0.009
Preterm birth < 37 weeks	17 (41.5%)	8 (47.1%)	9 (60%)	0.468
Delivery ≥ 37 weeks	20 (48.8%)	8 (47.1%)	5 (33.3%)	0.580
Delivery type				0.560
Vaginal delivery	17 (47.2%)	5 (31.25%)	6 (42.9%)	
Cesarean section	19 (52.8%)	11 (68.75%)	8 (57.1%)	
Complications	8 (19.5%)	2 (11.8%)	6 (40%)	0.133
PPROM	7 (17.1%)	2 (11.8%)	6 (40%)	0.101
Chorioamnionitis	1 (2.4%)	0	0	0.673

Data are presented as mean ± SD or n (%). PPROM, preterm premature rupture of membranes. ^a^*P* < 0.05, history-indicated group versus physical examination-indicated group. ^b^*P* < 0.05, ultrasound-indicated group versus physical examination-indicated group

Neonatal outcomes of all groups were demonstrated in Table 3. Five (12.2%) of the patients in history-indicated group, one (5.9%) of the patients in ultrasound-indicated group and three (20%) of the patients in physical examination-indicated group had abortions before the 22th week and/or newborn information could not be reached. Therefore, the results of these patients were not included in the statistical analysis of neonatal outcomes. Although not statistically significant, low birth weight (< 2500 g), low APGAR score, neonatal intensive care unit (NICU) admission and neonatal mortality rate were higher in physical examination-indicated group compared to history- and ultrasound-indicated groups (*p* > 0.05).

**Table 3:** Neonatal outcomes of the newborns according to cerclage indications

	**History-indicated cerclage (*n* = 36)**	**Ultrasound-indicated** **cerclage** (***n* = 16)**	**Physical examination-indicated** **cerclage (*n* = 12)**	***P*** **value**
Birth weight (g)	2500 ± 967	2645 ± 814	1912 ± 1232	0.129
LBW (< 2500 g)	13 (36.1%)	5 (31.2%)	8 (66.6%)	0.118
APGAR < 7 at 1st minute	10 (27.7%)	3 (18.7%)	7 (58.3%)	0.065
APGAR < 7 at 5th minute	5 (13.9%)	2 (12.5%)	4 (33.3%)	0.256
NICU admisson	15 (41.6%)	6 (37.5%)	7 (58.3%)	0.508
Neonatal mortality	3 (8.3%)	0	2 (16.6%)	0.262

Data are presented as mean ± SD or n (%). *LBW* Low birth weight, *NICU* Neonatal intensive care unit

## Discussion

In this study, we compared the results of cerclage procedures according to different indications. Our study demonstrated better pregnancy outcomes after history- and ultrasound-indicated cerclage than the physical examination-indicated cerclage. However, there was no statistically significant difference in terms of neonatal outcomes.

It is observed that the gestational week of cerclage placement for history-indicated group was significantly earlier compared to other groups. Since the preterm birth risk was determined from the obstetric history of the patients and cerclage was placed before any cervical change, the procedure was performed at earlier weeks for history-indicated group (16.1 ± 2.7 weeks). Although gestational age at cerclage was significantly higher for physical examination-indicated cerclage (20.6 ± 1.5 week), the mean duration from cerclage to delivery was also significantly lower for physical examination-indicated cerclage. It is also found that patients with history-indicated cerclage had statistically significantly longer cervix before the cerclage procedure (27.3 ± 6.9 mm, *p* < 0.001). The mean gestational week of delivery in patients with physical examination-indicated cerclage group was lower (31.7 ± 5.2 weeks) but not statistically significant. Gestational age at delivery in history-indicated group was 3.2 week longer than physical-indicated group but 1.2 week shorter than ultrasound-indicated group. Although cervical measurements of patients with ultrasound- (17.1 ± 3.4 mm) and physical examination-indicated cerclage (17.2 ± 6.3 mm) were similar, gestational age at delivery in ultrasound-indicated group was 4.4 week longer. Contrary to our study, many authors found that cervical length was significantly lower or even zero in pregnancies with physical examination-indicated cerclage [9–11]. However, Khan et al. evaluated the outcomes of pregnant women who underwent the history-, ultrasound- and physical examination-indicated cerclages. Similar to our study, they did not find any significant difference in the prevalence of short cervix (< 25 mm) between ultrasound- (38.5%) and physical examination-indicated cerclage (41.2%) groups [12]. This discrepancy between some other reports and our study could be explained by clinical characteristics of the patients in the physical examination-indicated cerclage group. We thought that most of our patients in the physical examination-indicated cerclage group had a cervix without significant dilatation. Supporting our view, Steenhaut et al. reported that cervical length was longer among patients undergoing emergency cerclage with non-prolapsed membranes compared to those with prolapsed membranes. They also stated that cervical dilation is more advanced when membranes are prolapsed beyond the external os [13].

Our findings demonstrated that the rate of preterm delivery was higher in cerclage cases with physical examination indications than those with history and ultrasound indications. Furthermore, all neonatal complications including low birth weight, low APGAR scores, neonatal intensive care unit admission and neonatal mortality were more common in physical examination-indicated cerclage. It has been known that physical examination-indicated cerclages were placed in emergency conditions with women presented with cervical dilation and prolapsed membranes. Data regarding physical examination-indicated cerclage due to cervical changes is limited. In a historical cohort study by Pereira et al., 225 patients with a dilated cervix were evaluated between 14 and 25 + 6 weeks of gestation. 152 received a physical examination-indicated cerclage and 73 were managed expectantly. They found that gestation period was longer, delivery rate was higher after 28 weeks and neonatal outcomes were observed better in physical examination-indicated cerclage than expectant management [7].

Although delivery ≥ 37 weeks were not significantly lower in our patients with physical examination-indicated cerclage, it was observed that they had significantly higher delivery rate < 28 weeks and < 34 weeks of gestation compared to those with history- and ultrasound-indicated cerclage. For this reason, all pregnant women should be considered in detail in terms of cervical insufficiency in the first trimester of pregnancy or at the beginning of the second trimester. If patients with a history of cervical insufficiency are determined, cerclage could be performed early for history indication rather than physical examination indication. Similar to our study, Chen et al. stated that the ultrasound-indicated group and history-indicated group were better in terms of pregnancy outcomes such as gestational age at delivery, APGAR scores and fetal survival rate compared with the physical examination-indicated group [10].

PPROM is the most frequently observed complication after cerclage [14]. It is possible to be seen during, immediately after or after a certain period of time. The probability of its occurrence in the whole pregnancy population is observed as 3%, and previous reports defined higher PPROM rates associated with cerclage. In the study conducted by Liu et al., PPROM complicated 30% and 39% of pregnancies after prophylactic and therapeutic cerclage, respectively [11]. However, a recent study by Muniz Rodriguez et al. found that all three types of cerclage procedures did not increase the risk of PPROM before 34 weeks when compared with pregnancies at increased risk for preterm birth [15]. They also attributed higher rates of PPROM reported previously among patients with cerclage to significant baseline risk for preterm birth due to dilated or short cervix, prior history of preterm birth or PPROM and prior cerclage. In this study, the incidence of PPROM in history- and ultrasound-indicated cerclage was found to be 17.1% and 11.8% which were substantially lower compared to physical examination-indicated cerclage (40%). The different rates of PPROM after cerclage placement could be related to different surgical techniques or different populations. Therefore, it would be appropriate to inform at least patients with physical examination-indicated cerclage for potential complications.

The beneficial effects of cerclage cases with history-related indications on perinatal and neonatal outcomes have also been shown in previous studies and similar results were found in this study [11, 16]. In a study conducted by Krispin et al. in 2019, they evaluated the cerclage results according to their indications and stated that the fetal complications that may occur due to preterm labor were observed more frequently in patients who underwent physical examination-indicated cerclage compared to history-indicated cerclage [16]. Korb et al. showed that cerclage reduces the birth rate before 24 weeks and perinatal mortality in cases with cervical shortening on ultrasound and a history of preterm birth [17]. In a meta-analysis including 15 studies, Alfirevic et al. showed the positive effect of ultrasound-indicated cerclage procedure on pregnancy outcomes [18]. Similarly, positive pregnancy outcomes were observed in our patients with ultrasound-indicated cerclage.

The retrospective nature of the study and the relatively small number of patients are the main limitations of this study. Furthermore, the absence of a control group in this study can be considered the most important obstacle in terms of generalizing the results. Finally, we were unable to measure some of the previously identified confounding factors for preterm birth including race, ethnicity, socioeconomic status, body mass index, uterine anomalies and maternal systemic morbidities. These factors might have affected our analyses. However, two-thirds of preterm births occur among women with no risk factors, causality has been difficult to prove and few interventions have been proven to prolong pregnancy in women at risk. Furthermore, our study groups did not have significant differences in many baseline characteristics. Therefore, the level of contribution of these factors and their subsequent impact on the present study was mitigated significantly. Strengths of our study include management of all patients with the same treatment protocols and with a uniform surgical technique in a tertiary center.

In conclusion, in this study, we investigated and compared the perinatal outcomes of cerclage procedure based on indications. Physical examination-indicated cerclage was associated with increased risk for preterm delivery < 28 weeks and < 34 weeks of gestation compared with history- and ultrasound-indicated cerclage. Therefore, consideration should be given to perform cerclage electively before the start of the process of preterm labor and in the early phase of cervical changes, rather than when the process of incompetence has already begun.

## Data Availability

The datasets used and/or analysed during the current study available from the C.G., H.G. or B.B. on reasonable request.
